# Use of prospective hospital surveillance data to define spatiotemporal heterogeneity of malaria risk in coastal Kenya

**DOI:** 10.1186/s12936-015-1006-7

**Published:** 2015-12-01

**Authors:** Donal Bisanzio, Francis Mutuku, Angelle D. LaBeaud, Peter L. Mungai, Jackson Muinde, Hajara Busaidy, Dunstan Mukoko, Charles H. King, Uriel Kitron

**Affiliations:** Department of Environmental Sciences, Emory University, Atlanta, GA USA; Department of Environment and Health Sciences, Technical University of Mombasa, Mombasa, Kenya; Department of Pediatrics, Stanford University, Stanford, CA USA; Center for Global Health and Diseases, Case Western Reserve University, Cleveland, OH USA; Vector-Borne Diseases Control Unit, Msambweni County Referral Hospital, Kwale, Msambweni, Kenya; Ministry of Health Kwale County, Kwale, Mombasa, Kenya; Vector-Borne Diseases Control Unit, Ministry of Health, Nairobi, Kenya; Department of Zoology, University of Oxford, Oxford, UK

**Keywords:** Malaria/epidemiology, Malaria/statistics and numerical data, Distribution/spatial, Public health surveillance, Spatiotemporal analysis, Geographic mapping, Geographic information systems, Kenya

## Abstract

**Background:**

Malaria in coastal Kenya shows spatial heterogeneity and seasonality, which are important factors to account for when planning an effective control system. Routinely collected data at health facilities can be used as a cost-effective method to acquire information on malaria risk for large areas. Here, data collected at one specific hospital in coastal Kenya were used to assess the ability of such passive surveillance to capture spatiotemporal heterogeneity of malaria and effectiveness of an augmented control system.

**Methods:**

Fever cases were tested for malaria at Msambweni sub-County Referral Hospital, Kwale County, Kenya, from October 2012 to March 2015. Remote sensing data were used to classify the development level of each monitored community and to identify the presence of rice fields nearby. An entomological study was performed to acquire data on the seasonality of malaria vectors in the study area. Rainfall data were obtained from a weather station located in proximity of the study area. Spatial analysis was applied to investigate spatial patterns of malarial and non-malarial fever cases. A space–time Bayesian model was performed to evaluate risk factors and identify locations at high malaria risk. Vector seasonality was analysed using a generalized additive mixed model (GAMM).

**Results:**

Among the 25,779 tested febrile cases, 28.7 % were positive for *Plasmodium* infection. Malarial and non-malarial fever cases showed a marked spatial heterogeneity. High risk of malaria was linked to patient age, community development level and presence of rice fields. The peak of malaria prevalence was recorded close to rainy seasons, which correspond to periods of high vector abundance. Results from the Bayesian model identified areas with significantly high malaria risk. The model also showed that the low prevalence of malaria recorded during late 2012 and early 2013 was associated with a large-scale bed net distribution initiative in the study area during mid-2012.

**Conclusions:**

The results indicate that the use of passive surveillance was an effective method to detect spatiotemporal patterns of malaria risk in coastal Kenya. Furthermore, it was possible to estimate the impact of extensive bed net distribution on malaria prevalence among local fever cases over time. Passive surveillance based on georeferenced malaria testing is an important tool that control agencies can use to improve the effectiveness of interventions targeting malaria (and other causes of fever) in such high-risk locations.

**Electronic supplementary material:**

The online version of this article (doi:10.1186/s12936-015-1006-7) contains supplementary material, which is available to authorized users.

## Background

The World Health Organization (WHO) estimates that almost 90 % of malaria-associated mortality occurs in endemic countries of sub-Saharan Africa [1]. Malaria is endemic in Kenya but, as is also the case in the rest of sub-Saharan Africa, transmission intensity has been drastically reduced since early 2000s [2, 3]. This decline is associated with an intensive anti-malaria campaign [4–7] based on massive distribution of long-lasting insecticide-treated bed nets (LLINs), indoor residual spraying (IRS) and the introduction of artemisinin-based combination therapy (ACT) as the first-line treatment for malaria [8, 9].

Malaria transmission patterns are modulated by the interactions between environmental, meteorological and socio-economic factors [10–13]. Spatial heterogeneity of malaria manifests in hot spots of transmission at different ranges of geographical scale [10, 14, 15]. Temporally, these hot spots show a seasonal pattern as well as inter-annual variability [11, 15]. Early detection and prediction of hot spots through an effective surveillance system can help target interventions aimed at reducing the impact of malaria in these areas.

Malaria hot spots have been identified by both passive and active surveillance systems in Kenya. Both systems can capture the space–time pattern of malaria and the impact of control systems on the disease’s morbidity [3, 16, 17]. However, in comparison to active surveillance, prevalence and incidence obtained by passive surveillance are more susceptible to population characteristics (e.g., education, wealth status) as well as distance from health care facilities [18, 19]. However, passive surveillance can cover a much larger area at a lower cost compared to active surveillance [15, 20], making it an important monitoring and evaluation tool for policymakers who chose to further enhance their control programs after an initial, but limited, reduction in malaria within endemic areas [3, 9, 21, 22]. In order to use data from health care facilities to estimate effectiveness of control programs, the passive surveillance system has to be based on an adequate testing system [23]. Following WHO guidelines [24], Kenya’s public health system has implemented a diagnosis-based malaria treatment policy for all age groups [9]. This policy has streamlined the collection of countrywide data that can be used to target areas with high resource needs for intervention.

Improved malaria testing practices at health care facilities in Kenya have indicated that a high proportion of febrile cases are not linked to *Plasmodium* infection [25]. Many diseases present in sub-Saharan Africa can manifest malaria-like symptoms, and only testing can lead health care practitioners to make correct diagnoses and subsequently prescribe the correct treatment [26]. While some of these diseases are well known and endemic in Kenya (e.g., influenza, pneumonia, enteric fevers), others are considered to be emerging (e.g., Rift Valley fever, chikungunya, dengue) [27–29].

In this study, data collected from one hospital located in coastal Kenya were used to: (1) calculate the fraction of fevers due to malaria; (2) describe the space–time pattern of malaria occurrence; (3) identify areas where non-malarial fever illnesses were more frequent; and, (4) assess the ability of passive surveillance to capture the short- and long-term effects of enhanced LLIN distribution for local populations at risk for malaria.

## Methods

### Ethical approval

Ethical approval and oversight for this study was provided jointly by the Institutional Review Board of the University Hospital Case Medical Center of Cleveland (Protocol 11-07-45) and by the Ethical Review Committee of the Kenya Medical Research Institute (KEMRI) (Non-SSC Protocol 087). The present analysis used aggregated, anonymized data reported to the investigators by the study health facilities as part of ongoing public health surveillance for malaria.

### Setting and data collection of incident febrile illnesses

The study was conducted in Msambweni sub-County Referral Hospital, Kwale County, Kenya (4.48°S, 39.48°E). The area is rural, and malaria is endemic, as are various other parasitic diseases [5–7, 10, 30]. The climate is characterized by monsoonal ‘long rains’ (April–June, LRS) and ‘short rains’ (October–December, SRS) rainy seasons, and by hot (January–March, HDS) and cool (July–September, CDS) dry seasons. Although rains are more frequent during the rainy seasons, rains also fall during the dry seasons. An extended bed net distribution program for all area households was implemented in the study area during August 2012 as part of the national malaria control program.

From October 2012 to March 2015, clinic-based surveillance of febrile cases was conducted at Msambweni Hospital. The hospital has 155 in-patient beds and serves as one of the main of health care providers in Kwale County. All patients presenting with fever (axillary temperature of 37.5 °C or above) or having history of fever were tested for malaria (*Plasmodium* spp. infection) using a standard, quality-controlled, Giemsa-stained, blood smear technique performed by trained parasitology technicians. For this study, only febrile illness cases positive by microscopy were counted as malaria diagnoses. Limited, fully anonymized data were provided by the hospital about patient age, gender and community of origin. Because patient identity was masked, the analyses could not be adjusted for repeated episodes of fever in the same person.

### Community characterization

Population and environmental characteristics were obtained for each community. Population size was based on the 2009 national Census [31]. Each community was characterized as ‘less-developed’ or ‘more-developed’, based on proportion of houses with thatched roof, spatial arrangement of households, and typology of road (road class and surface material), using high resolution satellite images from Google and Bing mapping systems acquired during 2006 and 2007, applying Quantum GIS (QGIS) [32] dedicated plug-ins. Information on the road network of the study area was gathered using data downloaded from the Global Roads Open Access Data Set website [33]. Google and Bing mapping systems were used to identify presence of rice fields adjacent to or within (≤1 km) of each community.

### Rainfall data

Historical weather data were obtained from October 2012 to March 2015 from the archive of Weather Underground website [34], recorded at the weather station located at the Moi International Airport of Mombasa (HKMO, 4.04°S, 39.59°E). The Moi International Airport of Mombasa is the closest weather station to the study area, located 56.6 km to the north of Msambweni Hospital.

### Entomological survey

Seasonal patterns of mosquito abundance were estimated using data obtained during a four-year (April 2009–April 2013), multi-village, entomological study in Kwale County [10, 35]. The entomological surveillance targeted four villages that were representative of communities of the south coast of Kenya, and the group of surveyed villages included two communities, Milalani and Nganja, that were part of the current study.

### Spatial analysis

**Fig. 1:**
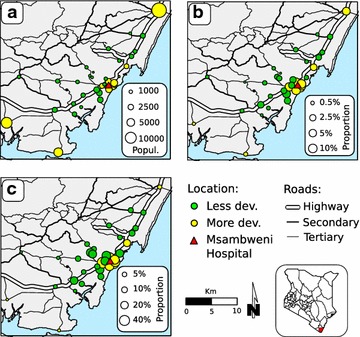
Characteristics and proportion of recorded febrile cases by community. **a** Village population (*circles*, where *circle* size indicates relative population size and *color* indicates level of development); **b** proportion of community population enrolled in the study as febrile cases at Msambweni Hospital (hospital indicated by *red triangle*, village *circle* size indicates relative proportion value); **c** proportion of admitted febrile cases from each community. Villages are categorized as less developed (*green*) or more developed (*yellow*). Road networks (major and minor roads) are also shown

Getis’ Gi*(*d*) local statistic [34] was applied to identify spatial clusters of high and low proportion of febrile cases associated with *Plasmodium* infection. Given the distribution of communities in the study area (Fig. 1), an automatic procedure (e.g., K nearest neighbors, distance threshold) could not be used to determine the distance weight for the Gi*(*d*) test. Instead, a neighboring network was created ad hoc, in which the links were based on Euclidian distance and road connections between villages (Additional file 1). Significance (p < 0.05) was evaluated by comparing expected values under the null hypothesis of complete spatial randomness (based on 999 Monte Carlo permutation) with observed data.

### Statistical modelling

Structured additive regression (STAR) models [33] were used to quantify the contribution of demographic attributes of patients, environmental characteristics of villages and seasonality to the probability of a febrile case being positive for *Plasmodium* infection. A STAR model was performed in order to account for spatial autocorrelation and seasonality of proportion of *Plasmodium* infection among febrile cases. The full model formula was:

$$\begin{aligned} Malaria \, case \, \left( {1,0} \right) = \, f_{1} \left( {Age} \right) \, + \, f_{2} \left( {Month} \right) \, + f_{3} \left( {LLINs \, distr.} \right) \, \hfill \\ + \, \beta_{1} * \, Gender \, + \, \beta_{2} * \, Dist \, Shore \, + \, \beta_{4} * \, Rice \, + \, \beta_{5} * \, \left( {Month* \, Rice} \right) \, \hfill \\ + B_{6} *\left( {Development} \right) \, + f_{spat} \left( {Communities} \right) + \, rand\,\left( {Communities} \right). \hfill \\ \end{aligned}$$

The model included four linear predictors: patient gender (*Gender*), distance from shoreline in km (*Dist Shore*), presence of rice fields nearby or within (≤1 km) the community (*Rice*), and community development level (*Development*). The model had a factor to represent the interaction between month and presence of rice fields (Month* Rice). Patient age and enrolment month were included as non-linear predictors (*f*_*1*_(*Age*), *f*_*2*_(*Month*)) modeled as natural cubic B-splines with a second-order random walk penalty. The effect of the mass distribution of LLINs performed in August 2012 was represented using a non-linear function (cubic B-spline) of the logarithm of number of months since the time between bed net deployment and each patient’s subsequent febrile episode (*f*_*3*_(*LLINs distr.*)).

The model contains a spatial correlated random effect, *f*_*spat*_ (*Communities*) modeled as a Markov random field. To describe the spatial relationship between villages, the neighboring network created ad hoc to perform the Getis’Gi*(*d*) was used (Additional file 1). The distance between each connected community was used as the weight of each network link. The model also had an unstructured random effect, *rand*(*Communities*), to consider heterogeneity among communities, that was not accounted by the model covariates, and adjusted the model for the distance (km) of community centre from the hospital.

Multivariate logistic regressions were used to estimate the associations of rice fields, seasonality and collection year with presence of female anopheline mosquitoes during each house collection session using a generalized additive mixed model (GAMM) [36] that took into account differences in sampling schedules between years (Additional file 2). Given the differences in sensitivity of sampling method applied during collection [35], each model was adjusted for the sampling techniques applied during each mosquito collection. The full model formula was:

$$\begin{aligned} Presence \, of \, Anopheles \, sp./Anopheles \, funestus/Anopheles \, gambiae \, \left( {1,0} \right) = \, f_{1} \left( {Month} \right) \, + \, \beta_{1} * \, Rice \, \hfill \\ + \, \beta_{2} * \, Year \, + \, rand \, \left( {Village} \right). \hfill \\ \end{aligned}$$

Collection month was included in the model as a non-linear predictor (*f*_*1*_(*Month*)). Presence of rice fields near or within the community (*Rice*) and collection year (*Year*) were included as linear predictors. The variable *rand*(*Village*) represents the random effect of the four villages where the collection was performed.

Multi-model selection approach based on Akaike Information Criteria (AIC) was performed to find the best models for the febrile illness data and the entomological data [37]. The ΔAIC was calculated among all proposed models as the difference between their AIC and the one with the lowest AIC value. All those models showing a ΔAIC <2 were included in the set of best models [37]. Presence of spatial and temporal autocorrelation in model residuals was tested using Moran’s I and Durbin-Watson test, respectively. Residuals of the entomological model were only tested for temporal autocorrelation due to the low number of sampled villages.

### Other statistical analysis

Association between distance from the Msambweni Hospital and the number of in-patients admitted from each community was tested using Spearman’s correlation. Fisher’s exact test was applied to evaluate differences of proportion of malaria febrile cases between females and males, and between less-developed and more-developed communities. The Fisher’s exact test was also applied to compare the proportion of houses positive to the presence of vectors between seasons. Proportions of febrile cases diagnosed with malaria were compared between age groups and seasons using Fisher’s least significant difference (HSD) test [38]. Wilcoxon rank-sum test was used to compare number of enrolled cases between seasons.

### Geographic information system and statistical tools

Data were stored in a geographic information system (GIS) created with QGIS software [39]. All geographic data were georeferenced using Universal Transverse Mercator (UTM) Zone 37 South, datum WGS84. Getis’Gi*(*d*) *test* was performed using Easyspat (Bisanzio et al. in prep.). Modelling was performed using the statistical software BayesX through the R software interface R2BayesX [33]. All other analyses and data cleaning were performed using basic functions embedded in R software [40].

## Results

### Village characteristics

Of the 34 villages included in the study, 24 (70.6 %) were classified as less developed (Fig. 1). Presence of rice fields was recorded in 21 communities (61.7 %), and most of these (18/21, 85.7 %) were classified as less developed.

The average distance from the shoreline and the average village elevation were 3.3 km (SD = 2.9) and 29.8 m (SD = 23.5) above sea level (masl), respectively. The median population size was 1698 [interquartile range (IQR) = 1052–2726] (Fig. 1a). The average distance of villages from Msambweni Hospital was 8.4 km (SD = 6.6) (Fig. 1); for each community, the proportion of all patients treated at Msambweni Hospital was negatively correlated to its distance from the hospital (Spearman’s ρ = −0.86, p < 0.01, Fig. 1c).

### Febrile cases

**Fig. 2:**
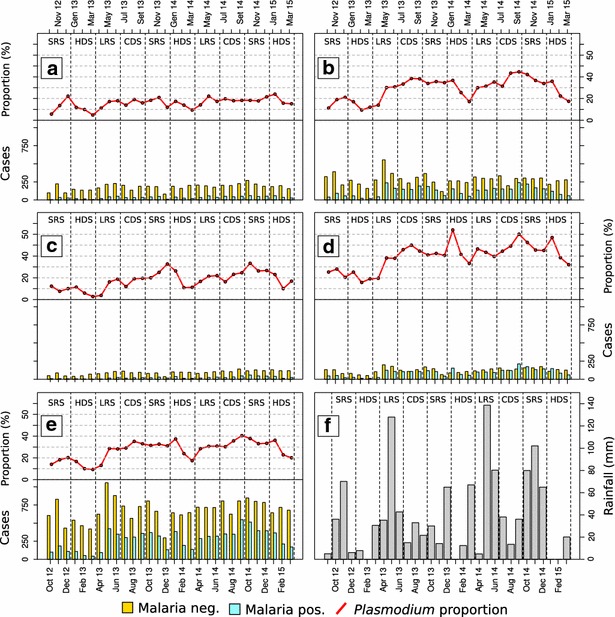
Monthly number of malaria-positive and -negative febrile cases, proportion of *Plasmodium* infections among febrile cases, and monthly cumulative rainfall. Figure panels show proportion of *Plasmodium* among febrile cases in children and in adults from less developed and more developed communities: adults (**a**) and children under 15 years (**b**) living in more developed areas; adults (**c**) and children (under 15 years); (**d**) living in less developed areas; data for all subjects enrolled in the study are shown in **e**. **f** Shows monthly rainfall recorded during the study period

Demographic characteristics and malaria prevalence of tested individuals are shown in Table 1. Over the study period, 25,779 febrile cases who sought health care at Msambweni County Referral Hospital were enrolled in the study. The median age of patients was 5 years (IQR = 2–21), with more females than males (Table 1). Overall, the blood smears of 7424/25,779 patients (28.7 %) were positive for *Plasmodium* species. Of all patients, 16,980 (65.8 %) were under 16 years of age, and the prevalence of malaria in this age group (34.7 %) was significantly higher than in adults (17.3 %, Fisher’s exact test, p < 0.01) (Fig. 2). Prevalence in male patients (32.4 %) was higher than in female patients (26.2 %, Fisher’s exact test, p < 0.01). Febrile cases from the less developed communities showed significantly higher prevalence of malaria (34.7 %) than those from the more developed villages (25.7 %, Fisher’s test, p < 0.01) (Fig. 2). However, among all less developed communities, those villages with rice fields within their borders or nearby had significantly higher malaria proportion (35.8 %) among enrolled febrile cases than less developed communities without rice fields (29.7 %, Fisher’s exact test, p < 0.01).

**Table 1:** Demographic characteristics of tested individuals having febrile illness, and their sub-group malaria prevalence by gender, age group and community type

	Sub-group proportion among the 25,779 patients tested (%)	Fraction of tested sub-group subjects found to have malaria (%)
Sex		
Female	58.4	26.2
Male	41.6	32.4
Age group		
0–5	46.9	28.7
6–10	12.5	49.8
11–15	6.1	44.3
16–20	6.2	24.3
21–25	7.4	18.8
26–30	6.3	16.1
>30	14.6	10.7
Type of community		
More developed	33.5	34.7
Less developed	66.5	25.7

**Fig. 3:**
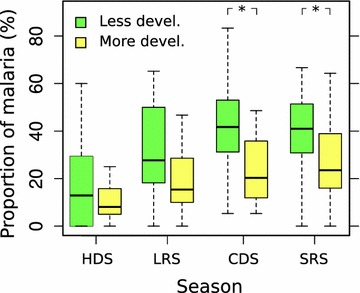
Boxplot of seasonal proportion of malaria infections among febrile cases by community typology. *Asterisk* indicates a significant difference between village categories (p < 0.05, Fisher’s exact test) by season

The number and proportion of enrolled febrile cases was associated with seasonality and showed two annual peaks during the LRS and during the SRS in 2013, and at the beginning of HDS and during SRS in 2014 (Fig. 2). This seasonal pattern was consistent for all febrile cases, both non-malarial and malaria-associated (Fig. 2). A significantly lower number of febrile cases was recorded during the HDS (Wilcoxon rank-sum test, p < 0.05). The seasonal trends of enrolled cases were similar in less developed and more developed communities (Fig. 2). The respective proportions of febrile cases with malaria were not significantly different between less and more developed communities during the HDS and the LRS (Fisher’s exact test, p > 0.05, Fig. 3). However, during the CDS and the SRS, a significantly higher (Fisher’s test, p < 0.05) proportion of febrile cases positive to malaria were from less developed communities (Fig. 3).

### Spatial analysis

**Fig. 4:**
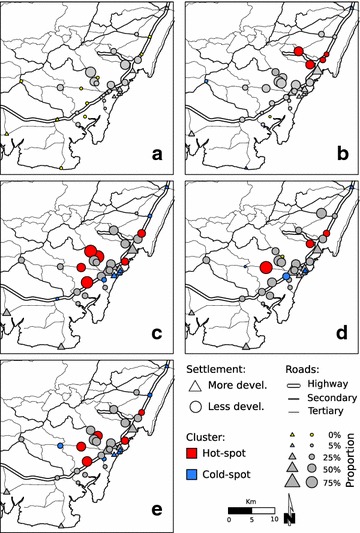
Getis *G*_*i*_ *(*d*) cluster analysis of study communities based on their higher or lower proportion of malaria-associated febrile cases. The *G*_*i*_ *(*d*) test was used to identify significant community hot-spot clustering (*red circles*) and/or cold-spot clustering (*blue circles*, p < 0.05, based on 9999 permutations) during: **a** the hot dry season (HDS, Jan–Mar); **b** the long rainy season (LRS, Apr–Jun); **c** the cool dry season (CDS, Jul–Sept); **d** the short rainy season (SRS, Oct–Dec); and, **e**, over all periods

The proportion of febrile cases who tested positive for malaria was spatially autocorrelated during the study period (Gi*(*d*) test, Fig. 4). Clusters of communities with high (hot spots) and low (cold spots) proportion of malaria-associated febrile illness were detected in every season except for the HDS. Most of the hot spots were around less developed communities and situated farther from the coast (Gi*(*d*) test, p < 0.05, Fig. 4). Low levels of malaria infections (cold spots) were clustered (*G*_i_*(*d*), p < 0.05, Fig. 4) around developed communities. No clusters were detected during HDS, when fewer *Plasmodium* infections were detected among patients coming from most of these communities (Fig. 4).

### Model results

**Fig. 5:**
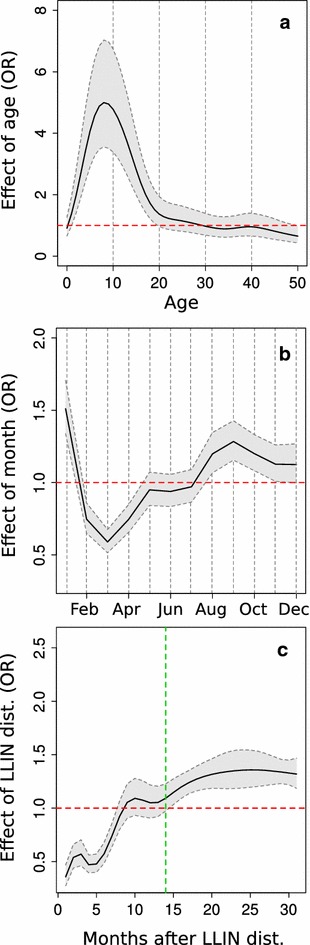
Age, month and time since last mass distribution of LLINs as non-linear predictors for the association of a febrile case with malaria infection obtained by the STAR model. **a** OR function of the age variable with 95 % CI; **b** OR function of the month variable with 95 % CI; **c** OR function of the time passed since mass distribution of LLINs with 95 % CI

**Fig. 6:**
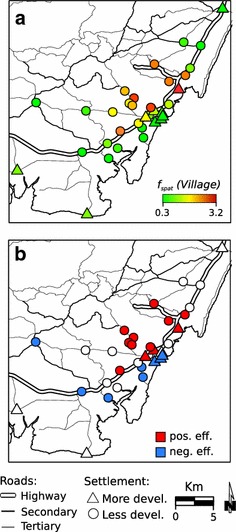
Estimated effect of spatial structured covariate *f*_*spat*_(*Village*) on the association of fever with malaria infection obtained by STAR model. **a** Mean OR for malaria-related fever in each community; **b** Communities showing a significant (p < 0.05) negative or positive effect in their proportion of *Plasmodium* infections among their febrile cases

The best model included variables of the full model formula (Additional file 3). Detailed results from the logistic STAR model are presented in Table 2 and Figs. 5 and 6. Males (Table 2), and children in the three to 18 years age range (Fig. 5) were significantly more likely to test positive for malaria. During the last 2 months of the HDS and first part of the LRS, patients were less likely to test positive for *Plasmodium* infection than in the CDS and SRS. Seasonal effects showed an interaction with presence of rice fields (Table 2).

**Table 2:** Predictors, based on logistic regression modelling, of the relative odds that a febrile case was associated with *Plasmodium* infection

Predictor	Value
	Odds ratio (95 % CI)
Linear fixed effect	
Sex: male	1.28 (1.12; 1.37)*
Distance to the shoreline (km)	1.10 (1.01; 1.24)*
Rice field presence	1.48 (0.71; 1.74)
No. months with rice field presence^a^	1.16 (1.08; 1.29)*
Less developed	1.15 (0.66; 1.99)
Smooth effect^b^	
Age	*
Month	*
Time since LLIN distribution	*
Random effect	
Village	Variance = 0.6
Structured spatial effect^c^	*

* p < 0.05; ** p < 0.01

^a^As counted from 1 to 12 months

^b^Only the significance of factors for age, month and time passed since mass distribution of LLINs is indicated; the smooth functions of these predictors are shown in Fig. 5

^c^Only the significance of structured spatial effect is indicated; the predictors are shown in Fig. 6

The mass distribution of LLINs in August 2012 was significantly associated with a reduced probability that febrile illness would test positive for malaria (Fig. 5c). However, the positive effect of the distribution of LLINs rapidly decreased and apparently disappeared by the 14th month post intervention (Fig. 5c).

Febrile patients from communities further from shoreline were more likely to test positive for *Plasmodium* infection (Table 2). The probability of a febrile case from less developed communities to have malaria was 50 % higher, but this result was not significant (Table 2). The spatial structure effect included in the model identified a hot spot of higher risk for malaria in the central part of the study area (Fig. 6), with patients from that area three times more likely to test positive for malaria. By contrast, patients from communities in the southern part of study area were significantly less likely to test positive for malaria (Fig. 6). No spatial (Moran’s I, p = 0.23) and temporal autocorrelation (Durbin-Watson test, p = 0.64) were found in model residuals. These findings showed that the model was able to capture the spatial–temporal component of the data.

### Mosquito infestation

During the study period, 2009–2013, 2463 households were surveyed for a total of 4125 house collections. Presence of female *Anopheles* mosquitoes was recorded in 461 households (18.7 %). The proportion of houses positive for presence of *An. funestus* and for *An. gambiae* were 11.4 % (282/2463) and 10.4 % (255/2463), respectively. Co-infestation by both species was recorded in 76 houses (3.1 %). The proportion of positive houses was higher during the rainy seasons (Fisher’s exact test, p > 0.05, Table 3).

**Table 3:** Seasonal mosquito collections and proportion of houses positive for female anopheline mosquitoes, 2009–2013

Study period (houses sampled)	*An. funestus*	*An. gambiae*	*Anopheles* genus	Co-infestation
All periods (2463)^a^	282 (11.4 %)	255 (10.4 %)	461 (18.7 %)	76 (3.1 %)
Season				
HDS (787)^b^	27 (3.4 %)	59 (7.5 %)	73 (9.7 %)	13 (1.7 %)
LRS (1132)^b^	114 (10.1 %)	109 (9.6 %)	198 (17.4 %)	23 (2.2 %)
CDS (1126)^b^	92 (8.1 %)	39 (3.4 %)	118 (10.4 %)	13 (1.2 %)
SRS (1080)^b^	76 (7 %)	69 (6.3 %)	128 (11.8 %)	17 (1.6 %)

^a^Number of unique houses sampled

^b^Number of house collections

### Model results for entomological collection

**Fig. 7:**
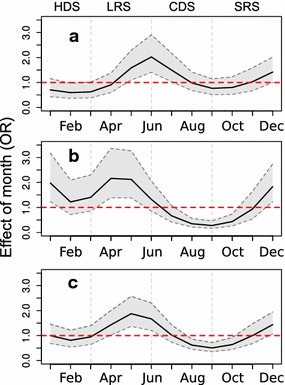
Month as non-linear predictor for presence of female *Anopheles* mosquitoes obtained by GAMM. **a** OR function of *An. funestus* presence; **b** OR function of *An. gambiae* presence; **c** OR function of either *Anopheles* species presence

Model selection results showed that all variable of the full formula were included in the best model (Additional file 4). Presence of female *Anopheles* mosquitoes showed significant seasonality, with the risk of mosquito infestation higher during rainy seasons (Fig. 6). The probability for detection of both *An. gambiae* and of *An. funestus* infestations was significantly higher in the LRS and lower in the CDS (Fig. 7). The probability for houses to be infested with *Anopheles* mosquitoes was significantly lower in 2009, the first year of collection (Table 4). Model results also indicated an association of presence of rice fields near a village with higher probability of infested houses, but this effect was not significant (Table 4). Model residuals did not show temporal autocorrelation (Durbin-Watson test, p = 0.37).

**Table 4:** Predictors for presence of female anopheline mosquitoes based on multivariable logistic regression modelling

Predictors	*An. funestus*	*An. gambiae*	*Anopheles* genus
Fixed effect	OR (95 % CI)	OR (95 % CI)	OR (95 % CI)
Presence of rice fields	1.1 (0.1–8.1)	3.1 (0.8–52.1)	2.4 (0.3–15.1)
Years	0.7 (0.6–0.8)**	0.7 (0.6–0.8)**	0.7 (0.6–0.8)**
Month^a^	*	*	*

Random effect	Variance	Variance	Variance
Village	1.1	3.3	1.5

The models were adjusted for the collection technique applied in each mosquito collection

* p < 0.05; ** p < 0.01

^a^Only the significance of month covariate is indicated; the smooth function of this predictor is shown in Fig. 7

## Discussion

Fever is the most common symptom exhibited by people seeking health care in Kenya [41–43]. The study results demonstrated that georeferenced information obtained through testing febrile cases for malaria can be used to evaluate the spatial and temporal heterogeneity in patterns of *Plasmodium* infection in a district-level or sub-county sized study area. Additionally, these data allowed associating a significant effect on the prevalence of malaria among febrile cases with mass deployment of LLINs, (which occurred during the summer of 2012). Finally, the study findings highlighted the presence of clusters of low prevalence of malaria in febrile cases in communities closer to the Indian Ocean shoreline.

The prevalence of malaria among febrile cases increased following the start of the two rainy seasons, when the frequent rains likely increased the abundance of breeding sites for *Anophele*s mosquitoes. This effect was more marked in the less developed communities, and could also be statistically linked to the presence of nearby rice paddies. In coastal Kenya, past research has indicated that rice fields become flooded during the rainy seasons and become optimal breeding sites for *Anopheles* mosquitoes for several months thereafter [44, 45]. The flooding period of paddies typically results in an increase of vector abundance in adjacent communities, likely followed by higher levels of transmission levels of *Plasmodium* spp. [46].

The seasonal trend of malaria prevalence among febrile cases was significantly associated with mosquito infestation levels recorded in sampled households. A significant reduction was observed in the proportion of infested houses from 2009 to 2013. This can probably be linked to the mass LLIN distribution campaign performed in the study area [47], as similar control efforts have demonstrated a decrease in infestation levels elsewhere [48, 49]. The proportion of fever cases who tested positive for malaria decreased significantly in the months following the mass deployment of LLINs. However, the observed reduction lasted less than 18 months after the distribution of LLINs. These findings are consistent with the previously recorded mean time of effectiveness of LLINs in coastal Kenya [50]. Moreover, studies have shown that bed net use declines after approximately 1 year of utilization as the bed nets are perceived as less effective due to accumulated damage [51–53].

Spatial analysis identified geographical hot spots of malaria risk in the central portion of the study area. The same areas were also indicated as being at high risk by the STAR model, which simultaneously accounted for the presence of rice fields, seasonality and each community’s level of development. Model results suggested the presence of additional factors not included as predictors in the performed model that may also play an important role in the spatial heterogeneity of malaria prevalence in surveyed populations. For instance, the model did not include information regarding the socio-economic status of enrolled individuals or other information concerning potentially important larval sites, such as the presence of permanent or seasonal ponds. These factors could have further affected the spatial heterogeneity of malaria prevalence. Such breeding sites (flooded, wet areas) increase the risk of infection in the surrounding communities and, accordingly, malaria hot spots are often identified near these areas [10, 54]. High malaria levels in poor communities can be attributed to the natural materials used to build houses (mud walls and grass-thatched roof), which provide optimal resting places for mosquitoes [55, 56]. However, the aforementioned environmental risk factors can also be found in some more developed communities, and these can increase the risk of malaria for people living in these areas as well [57]. This could explain why the STAR model identified some more developed communities as being at high risk of infection as well.

With regard to age, individuals aged three to 18 years showed a high probability of testing positive for malaria, and individuals 9 years of age had the highest estimated probability of malaria-positive fever. These results are consistent with previous findings based on active surveys performed in communities of the study area [10]. Additional studies in Uganda and Western Kenya [15, 20] have also shown the effectiveness of passive surveillance in identifying age groups that should be targeted by control systems.

Collection of data at health care facilities is more cost-effective than testing for malaria at the community level and can be easily maintained year round [15]. However, these types of data only include those community members who have sought medical attention. Information on malaria prevalence obtained by screening febrile cases cannot be used for accurate estimations of the true malaria prevalence in communities [58, 59]. Nevertheless, the analyses demonstrated that data recorded at health facilities can be used to determine those areas where *Plasmodium* circulation is very high. Studies performed in Rwanda have shown that active surveillance informed by data previously collected by passive surveillance can be used to analyse malaria hot spots and identify the likelihood of asymptomatic cases at community level [54].

Among the tested febrile cases, 28.5 % tested positive for malaria, which indicates that a wide proportion of febrile cases were due to other causes. Similar low malaria prevalence among febrile cases has been reported in other endemic countries of sub-Saharan Africa [25, 26, 60, 61]. In the study area, fever symptoms recorded in adults and inhabitants of more developed communities were more likely to be linked to non-malaria infections. Community clusters with high non-malaria febrile illness were found close to the coastline where levels of malaria prevalence are low [10]. Several prospective studies have found that febrile cases are often due to bacterial or viral diseases that mimic symptoms of malaria (e.g., dengue, chikungunya, leptospirosis, ehrlichiosis, brucellosis, enteric fevers) [25, 26]. New, emerging diseases are often misdiagnosed as malaria because they have similar symptoms, and this may be especially common for individuals with dengue fever who are, in practice, often treated with anti-malarials but without benefit [28, 62].

## Conclusions

The results obtained from data recorded at the Msambweni Hospital allowed describing temporal and spatial of malaria risk. These findings also suggested that passive surveillance can be an effective and low-cost method to monitor the impact of mass LLIN distribution. This information can be used by surveillance and control agencies for more effective targeting of interventions based on LLIN distribution or IRS. Notably, the study results highlighted that the majority of fevers in coastal Kenya were not linked to smear-positive malaria. Improved testing for proper diagnosis of febrile cases at health care facilities could further define geographical hot spots and seasonality of these other competing causes of life-threatening and disabling infections, and, consequently, allow health systems to apply better, cause-specific control.

## Additional files

10.1186/s12936-015-1006-7 The spatial structure used in the STAR model was the one used to perform the Gatis’*Gi*(d)*,
10.1186/s12936-015-1006-7 Timing of mosquito sampling sessions per each village during study period 2009-2013 (Figure).
10.1186/s12936-015-1006-7 Results of model selection performed to find the best model used to analyse febrile illness data (Table).
10.1186/s12936-015-1006-7 Results of model selection performed to find the best model used to analyse entomological data (Table).

## Abbreviations

LLINs: long-lasting insecticide-treated bed nets
ACT: artemisimin-based combination therapy
LRS: ‘long rains’ season
SRS: ‘short rains’ season
HDS: hot dry season
CDS: cool dry season
STAR: structured additive regression
GAMM: generalized additive mixed model
WHO: World Health Organization
IRS: indoor residual spraying
AIC: Akaike information criteria
