# Supplementary material for: Use of prospective hospital surveillance data to define spatiotemporal heterogeneity of malaria risk in coastal Kenya
Source: Malar J. 2015 Dec 1;14:482. doi: 10.1186/s12936-015-1006-7 (PMC4665820; doi:10.1186/s12936-015-1006-7)
Supplement: Supplementary file 1 — 10.1186/s12936-015-1006-7 The spatial structure used in the STAR model was the one used to perform the Gatis’Gi*(d), [file 12936_2015_1006_MOESM1_ESM.pdf]

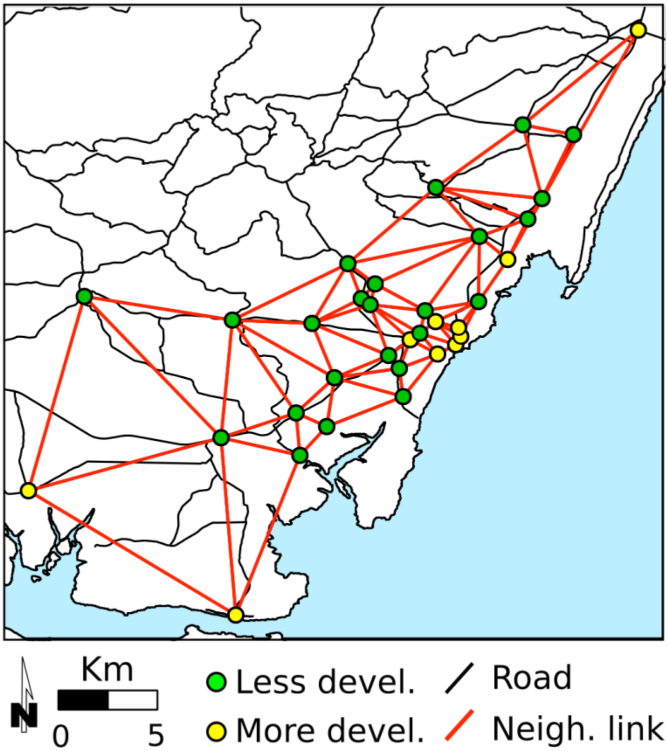

Additional file 1. The spatial structure used in the STAR model was the one used to perform the Gatis' *Gi*(d)*. The neighbour network was created *ad hoc* to capture the spatial proximity of communities. The links generated are based on distance and road connection between villages. Given the distribution of communities in the study area, an automatic function could not be used (e.g., K nearest neighbours, distance threshold) to create distance spatial weight for use in the *Gi*(d)* test. In the STAR model, each network link was weighted by the distance between the settlements of the dyad.
