# Supplementary material for: Use of prospective hospital surveillance data to define spatiotemporal heterogeneity of malaria risk in coastal Kenya
Source: Malar J. 2015 Dec 1;14:482. doi: 10.1186/s12936-015-1006-7 (PMC4665820; doi:10.1186/s12936-015-1006-7)
Supplement: Supplementary file 2 — 10.1186/s12936-015-1006-7 Timing of mosquito sampling sessions per each village during study period 2009-2013 (Figure). [file 12936_2015_1006_MOESM2_ESM.pdf]

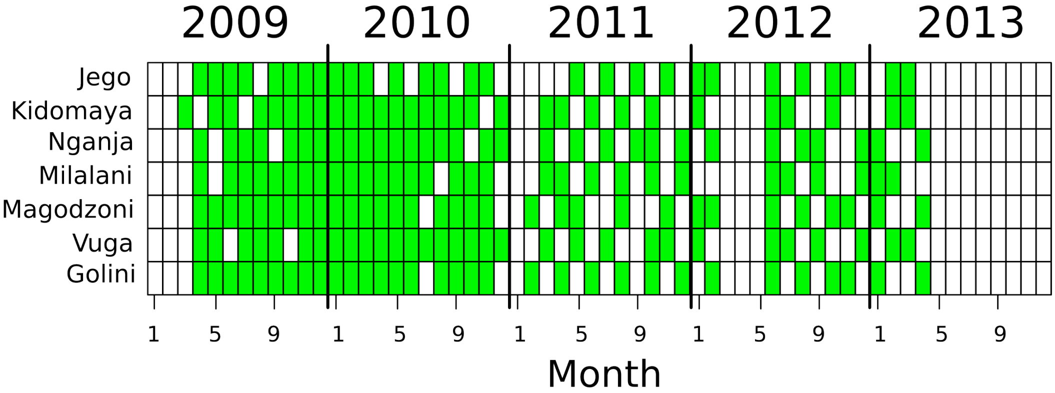

Additional file 2. Timing of mosquito sampling sessions per each village during study period 2009-2013. Green colour indicates months in which mosquito sampling was performed.
