# Supplementary material for: Use of prospective hospital surveillance data to define spatiotemporal heterogeneity of malaria risk in coastal Kenya
Source: Malar J. 2015 Dec 1;14:482. doi: 10.1186/s12936-015-1006-7 (PMC4665820; doi:10.1186/s12936-015-1006-7)
Supplement: Supplementary file 3 — 10.1186/s12936-015-1006-7 Results of model selection performed to find the best model used to analyse febrile illness data (Table). [file 12936_2015_1006_MOESM3_ESM.pdf]

Additional file 3 Results of model selection performed to find the best model used to analyse febrile illness data. The table only reports the first six tested models.

Model	*Gender*	*Dist shore*	*Rice*	*Development*	$f_1(Age)$	$f_2(Month)$	$f_3(LLINs\ distr.)$	$f_{spat}(Com.)$	*rand(Com.)*	*(Month* Rice)*	AIC	ΔAIC
1	+	+	+	+	+	+	+	+	+	+	29252.1	0
2	-	+	-	+	+	+	+	+	+	-	29248.4	3.7
3	-	-	+	-	+	+	+	+	+	-	29246.8	5.3
4	+	+	+	-	+	+	+	+	+	-	29244.7	7.4
5	+	+	+	-	-	+	-	+	+	+	29243.2	8.9
6	-	+	-	+	+	-	+	+	+	-	29241.3	10.8

'+'= variable included; '-'=variable dropped
