# Supplementary material for: Use of prospective hospital surveillance data to define spatiotemporal heterogeneity of malaria risk in coastal Kenya
Source: Malar J. 2015 Dec 1;14:482. doi: 10.1186/s12936-015-1006-7 (PMC4665820; doi:10.1186/s12936-015-1006-7)
Supplement: Supplementary file 4 — 10.1186/s12936-015-1006-7 Results of model selection performed to find the best model used to analyse entomological data (Table). [file 12936_2015_1006_MOESM4_ESM.pdf]

Additional file 4 Results of model selection performed to find the best model used to analyse entomological data. The table only reports the first six tested models.

Model	*Rice*	*Year*	f_1*(Month)*	*rand(Village)*	AIC	ΔAIC
1	+	+	+	+	11546.3	0
2	+	-	+	+	11542.1	4.2
3	-	+	+	+	11538.4	7.9
4	-	+	+	+	11533.4	12.9
5	-	-	+	+	11531.5	14.8
6	+	+	-	-	11525.2	21.1

'+'= variable included; '-'=variable dropped
